# The perspective of women with an increased risk of OHSS regarding the safety and burden of IVF: a discrete choice experiment

**DOI:** 10.1093/hropen/hoz034

**Published:** 2020-02-24

**Authors:** S C Braam, J P de Bruin, B W J Mol, M van Wely

**Affiliations:** 1 Centre for Reproductive Medicine, Department of Obstetrics and Gynaecology, Meibergdreef 9, 1105 AZ Amsterdam UMC, University of Amsterdam; 2 Centre for Reproductive Medicine, Department of Obstetrics and Gynaecology, Jeroen Bosch Hospital, ‘s-Hertogenbosch, The Netherlands; 3 Department of Obstetrics and Gynaecology, Monash University, Clayton, VIC, Australia

**Keywords:** discrete choice experiment, patient preference, ovarian hyperstimulation syndrome, gonadotrophins, IVM, IVF

## Abstract

**STUDY QUESTION:**

What are the preferences of women with an increased risk of ovarian hyperstimulation syndrome (OHSS) for characteristics of IVF treatments?

**SUMMARY ANSWER:**

In women with increased risk of OHSS, the chance of OHSS is a strong attribute in determining women’s preference for IVF treatment and women are willing to trade off burden (side effects), costs and chance of pregnancy for lower risks of OHSS.

**WHAT IS KNOWN ALREADY:**

OHSS is the most serious iatrogenic complication of ovarian stimulation. Polycystic ovaries, high antral follicle count (AFC) and previous OHSS increase the risk of developing OHSS. IVM of oocytes offers great potential for patients with high AFC, since there is no risk of OHSS. With regard to patients’ perspectives on fertility treatments, it has been shown that women undergoing IVF place different values on treatment characteristics, such as effectiveness (pregnancy rate), cancellation risk, safety (OHSS risk) and burden (side effects). To our knowledge, the preferences for different IVF treatments in women with increased risk of OHSS have not been studied yet.

**STUDY DESIGN, SIZE, DURATION:**

A multicentre discrete choice experiment (DCE) was performed between 2012 and 2016. The selected attributes offered were chance of OHSS, which represents safety; number of injections; chance of cycle cancellation (the latter two represent burden); chance of pregnancy; and out-of-pocket costs/willingness to pay. A target sample size was calculated by including 20 patients for five attributes resulting in the aim to include 100 women.

**PARTICIPANTS/MATERIALS, SETTING, METHODS:**

We invited subfertile women who were diagnosed with normogonadotrophic ovulation disorder and were undergoing treatment with gonadotrophins and/or had experienced (imminent) OHSS in a previous IVF treatment in the fertility clinic of four hospitals (three teaching and one academic). Women received a printed questionnaire with fictional scenarios and were asked, for each scenario, to choose their preferred treatment. We used a multinominal logit model to determine the preferences of women and investigated heterogeneity in preferences through latent class analysis. The decrease in OHSS risk required for women to accept an increased level of an undesirable attribute, i.e. their willingness to trade off, was calculated.

**MAIN RESULTS AND THE ROLE OF CHANCE:**

We distributed 120 questionnaires with a response rate of 79% (95/120). There were 91 questionnaires included in the analysis. All five attributes influenced women’s treatment preference. About half of the women considered chance of pregnancy to be more important, while the other half considered prevention of OHSS and lower costs to be more important. Women were willing to trade off cancellation rate, number of injections, chance of pregnancy and costs for lower OHSS chances. We found that women were willing to accept 5% more chance on cycle cancellation if the OHSS rate dropped with 2%. Women were willing to accept one extra treatment for a reduction of 3.9% in OHSS risk. With respect to costs, women were willing to pay €1000 instead of no costs for a decrease in OHSS rate of 5.4%**.**

**LIMITATIONS, REASONS FOR CAUTION:**

The sample size of our study is relatively small which may limit the generalizability and sensitivity of the study.

**WIDER IMPLICATIONS OF THE FINDINGS:**

The results of this DCE help us to understand the trade-off that women at risk of OHSS make in their preference for characteristics on IVF treatments. This knowledge may be used during the counselling of couples about their treatment options.

**STUDY FUNDING/COMPETING INTEREST(S):**

B.W.M. is supported by a NHMRC Practitioner Fellowship (GNT1082548). B.W.M. reports consultancy for Merck, ObsEva and Guerbet. J.P.d.B. reports personal fees from the Ferring Medical Advisory Board and grants from Ferring B. V and Merck Serono B. V outside the submitted work. There are no other conflicts of interest to declare.

**TRIAL REGISTRATION NUMBER:**

None.

**WHAT DOES THIS MEAN FOR PATIENTS?**

In IVF, fertility medications are given to women to make their ovaries produce mature eggs and it allows several eggs to be taken from the ovaries. Ovarian hyperstimulation syndrome (OHSS) is a serious medical issue that happens to some women, as a result of stimulating the ovaries in this way. OHSS is usually rare, but some women are more likely to suffer from it, for example women with polycystic ovary syndrome and women that have had OHSS before. An alternative to IVF is in vitro maturation (IVM), a similar fertility treatment with no risk of OHSS.

The researchers wanted to know what the views are of women who might be more likely to suffer from OHSS, on both treatments. What do women prefer, and what may influence their preference?

We invited these women to complete a questionnaire. For each question, the women could choose between two options. The questions asked about things like the chance of getting OHSS, number of hormone injections, cancellation of treatment cycles, cost and the chance of getting pregnant.

Ninety-one (91) women completed the questionnaire. Women who might be more likely to get OHSS thought that the possibility of getting OHSS was very important when deciding which fertility treatment they would prefer. About half of the women thought it was worth trading cancellation rate, number of injections, chance of pregnancy and costs for a lower chance of getting OHSS.

The researchers suggest that this research could be used by fertility specialists during the counselling of couples about their treatment choices.

## Introduction

IVF is a cornerstone in the management of subfertility. An important part of IVF treatment is ovarian stimulation. Ovarian hyperstimulation syndrome (OHSS) is the most serious iatrogenic complication of ovarian stimulation and is life-threatening in its severe form. There are large variations in the reported incidence of OHSS in IVF, with an estimated prevalence of 20–33% in its mild form and 3–8% in its moderate or severe form (Mourad *et al*. 2017). Polycystic ovaries with or without polycystic ovary syndrome (PCOS), a high antral follicle count (AFC) (e.g. at a young age) and previous OHSS increase the risk of developing OHSS (Delvigne *et al*. 1993; Humaidan *et al*. 2010). Early OHSS is an acute consequence of the exogenous hCG administration before oocyte retrieval and is usually related to an excessive ovarian response to gonadotrophin stimulation (Navot *et al*. 1992; Mathur *et al*. 2002). The only guaranteed method for prevention of early OHSS is to cancel cycles and withhold hCG. However, most physicians are reluctant to cancel a cycle, particularly in IVF where the financial burden of treatment and the patient’s psychological distress may be significant (Humaidan *et al*. 2010). Preventative strategies that appear highly effective at reducing or preventing OHSS include GnRH antagonist protocols and the use of GnRH agonists to trigger final oocyte maturation. Furthermore, IVM of oocytes offers great potential for patients with high AFC. IVM is one form of ART which involves the retrieval of multiple immature oocytes from the ovaries with minimal, or without any, gonadotrophin injections. Subsequently, these oocytes are matured *in vitro*. Since IVM does not require ovarian stimulation, the risk of OHSS is abolished while clinical pregnancy rates seem acceptable. Centres with experience of the technique have reported live birth rates of ~40% per embryo transfer (Junk and Yeap, 2012; Walls *et al*., 2015).

With regard to patients’ perspectives on fertility treatments, it has been known since the early 1990s that an IVF treatment is accompanied by mental and psychological distress (Kopitzke *et al*., 1991; Edelmann *et al*., 1994). Subfertile women have been noted to score higher on anxiety and depression scales before and during IVF treatment (Verhaak *et al*., 2005; Ying *et al*., 2016). Also, when looking at both burden and benefits of a treatment, several studies have shown that patients’ perspectives can differ from those of the health professionals (Devereaux *et al*., 2001). It has been shown that women undergoing IVF place different values on treatment characteristics, such as effectiveness (pregnancy rate), cancellation risk, safety (OHSS risk) and burden (side-effects) (van den Wijngaard *et al*., 2014; van den Wijngaard *et al*., 2015).

Studies assessing the impact of OHSS and cycle cancellation in women with an increased risk of OHSS are lacking.

We therefore performed a discrete choice experiment (DCE) in which we examined the preference of patients with an increased risk of OHSS for characteristics of IVF treatment and their evaluation of the risk of OHSS and cycle cancellation.

## Materials and Methods

We studied subfertile women who visited the fertility clinic of three teaching hospitals and one academic hospital in the Netherlands (Jeroen Bosch Hospital in ‘s-Hertogenbosch, Maxima Medical Centre in Veldhoven, Isala Clinics in Zwolle and Academic Medical Center in Amsterdam) between 2012 and 2016. Permission from the Jeroen Bosch Hospital’s Medical Ethics Committee was obtained. All women were referred for unfulfilled child-wish by a general practitioner, in general after failure to conceive despite 6–12 months of unprotected intercourse.

The following women with increased risk to develop OHSS were eligible: women with a normogonadotrophic ovulation disorder (PCOS) and undergoing treatment with gonadotrophins (ovulation induction (OI) with or without IUI); women who had developed OHSS in a previous OI or IVF treatment cycle; and women whose previous OI or IVF treatment cycle was cancelled due to imminent OHSS.

**Figure 1:**
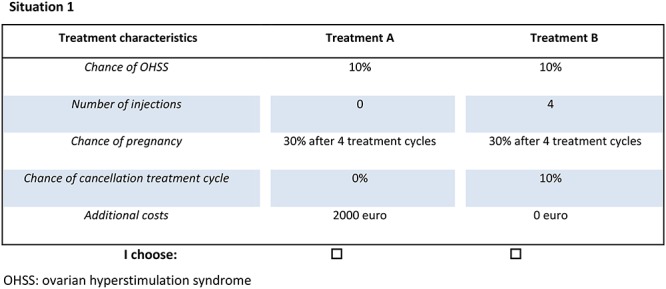
Example of discrete choice question in the discrete choice experiment questionnaire.

After informed consent, women received a printed questionnaire with 32 fictional scenarios, presented in 16 questions. Each question consisted of two fictional treatment options. Women were asked, for each scenario, to choose their preferred treatment (Fig. 1).

### Setting

In the Netherlands, every Dutch citizen has a mandatory basal level of health care insurance. The costs of this health insurance consist of the monthly premium and the ‘own risk’ amount, which is an annual amount that individuals must pay out of their own pocket for some treatments and medicines before the health insurance will cover the rest. In 2018, the ‘own risk’ was up to a maximum of 385 euros. The basal health insurance covers most primary and hospital care including all treatment and medication costs for OI, IUI and a maximum of three cycles of IVF/ICSI. After a successful IVF/ICSI pregnancy, another three cycles of IVF/ICSI are reimbursed.

### DCE

The DCE method is used to measure preferences in the face of multiple benefit–risk trade-offs. The design of this study was based on the recommendations of the International Society of Pharmacoeconomics and Outcomes Research (ISPOR) for good research practices for conjoint analysis (Reed Johnson *et al*., 2013; Hauber *et al*., 2016). A DCE assumes that a given treatment can be described by its characteristics or ‘attributes’. The patients’ preference for a treatment is determined by the variants or ‘levels’ of these attributes. The relative importance of the attributes and trade-offs that patients make between them can be assessed by offering a choice between several sets of alternatives with different combinations of attribute levels.

The selection of attributes and levels was based on the expert opinions of a focus group consisting of gynaecologists working in one of the participating hospitals who are specialized in fertility care. Furthermore, we interviewed women with an ovulation disorder and experience in different fertility treatments, including injections with gonadotrophins. We selected the following five attributes: chance of OHSS, which represents safety, daily s.c. injections and chance of cycle cancellation, which both represent burden, chance of pregnancy measured as number of treatments required to conceive, which represents benefit, and out-of-pocket costs/willingness to pay. The chosen attribute levels were based on data from the literature and the focus group. A summary of the attributes and their levels is shown in Table I.

**Table I:** Attributes and levels used in the discrete choice experiment design.

**Attribute**	**Levels**
Chance of OHSS Injections Chance of pregnancy Chance of cycle cancellation Contribution	0% 5% 10% 0 4 20 30% after 1 treatment 30% after 2 treatments 30% after 3 treatments 30% after 4 treatments 0% 5% 10% 15% None €1000 €2000 €3000

OHSS: ovarian hyperstimulation syndrome.

With regard to the attribute ‘injections’, level 20 injection was chosen based on the average number of injections needed for IVF treatment. Since different IVM techniques are applied with regard to the administration of hCG and recombinant FSH (rFSH) priming over 3 days, we chose the level 0 injections as well as four injections.

With regard to the attribute ‘contribution’, worldwide public financing of ART ranges from no reimbursement or funding a limited number of cycles to unrestricted reimbursement with co-payments. Therefore, the levels of ‘contribution’ were none (reimbursement) up to 3000 euro (out-of-pocket costs).

### Development of the choice sets

The combination of five attributes with their levels provided a total of 576 (3^2^ × 4^3^) possible scenarios (Table I). A functional sample of scenarios was selected using an orthogonal design (Louviere, 2007). This resulted in 32 scenarios, which were randomly combined into 16 discrete choice sets. Among the 16 questions, two represented dominance tests (rationality tests) as control. Dominance tests are questions in which one of the scenarios has all favourable attribute levels, so the answer will be predictable. Data from women who failed this rationality test were excluded from further analyses because they failed to understand the questionnaire.

### Questionnaire

The first part of the questionnaire consisted of general questions regarding baseline characteristics, i.e. age, educational level, duration of subfertility and previous experience with fertility treatment. There was also one open-ended question for the women to endorse their answers and add comments. Before answering the 16 choice sets, women received contextual information about the topic, attributes and task instructions, including a neutral written description of IVM and OHSS. Information was given about the risks and consequences of OHSS and its diverse appearances, such as nausea, pain and, as an ultimate consequence, death. At the end of the DCE, women were asked how easy or difficult they found the DCE using a scale from 1 (extremely difficult) to 10 (extremely easy). Before starting the study, the questionnaire was tested by a panel of doctors, nurses and patients to assess interpretation. A target sample size with the use of five attributes was calculated by using a rule of thumb of 20 patients per attribute for the main analysis. Since our DCE contained five attributes, we aimed to include 100 women.

### Statistical analyses

For demographic data, we calculated the mean and SD for continuous parameters and numbers and percentage for dichotomous or nominal data.

We used a main-effects multinominal logit model to determine the importance that women placed on each attribute and its levels (Hazlewood *et al*., 2016). The model assumed the probability of a participant choosing a given treatment within the set of choices to be related to an overall value (utility) of each treatment plus a random error. The overall value of the treatment was defined as the sum of the importance scores for the attribute levels which define the treatment. The attributes were primarily included as categorical variables, then as continuous variables after confirming a linear relationship through visual inspection and by comparing the Akaike information criterion (AIC) between models.

The output of the multinominal logit model includes mean coefficients representing the relative utility of each attribute conditional on other attributes and SDs of the random coefficients, along with their respective CIs. The negative sign of the coefficient reflects a negative effect on utility. The value indicates the relative importance of the attribute to total relative utility. Absolute values of the dependent variable and coefficients, however, were considered to have no direct interpretation (Louviere 2007). We also determined the willingness to trade off, i.e. the acceptance of an increased level of an undesirable attribute for a decrease in OHSS risk [marginal rate of substitution (MRS)]. The MRS was calculated by dividing the difference in the importance scores between the highest and lowest attribute levels by the importance of a major symptom improvement, modelled as a continuous variable. The median and 95% CIs of the MRS were estimated through Monte Carlo sampling (Berg, 2004).

Preference heterogeneity was investigated through latent-class analysis (LCA). With LCA, one can study whether groups of patients make comparable preference choices. This analysis allows one to estimate classes to which the participant most likely belongs. We fitted latent-class solutions with two and three classes, comparing measures of model fit (adjusted Bayesian information criterion and consistent AIC) and patterns of importance scores between models and to the overall multinomial logit model. Patients were assigned to the latent class for which they had the highest probability. We determined the association between selected patient characteristics and latent-class membership using univariate and multivariable logistic regression models. Multivariable models were considered exploratory and were limited to a maximum of two variables to avoid overfitting. Age and experience with having had a cycle cancelled were included a priori in view of their expected preference effect to these attributes on choice making. We only evaluated other variables when, on the basis of the results of univariate statistics, a variable was associated with preference at a *P* value <0.15. All analyses were performed using SPSS 24 (IBM: Armonk, NY, USA) and R (version 3.1.2; http://www.r-project.org).

## Results

### Participants

Out of the 120 women who agreed to participate, 25 did not return the questionnaire leading to a response rate of 79% (95/120). Three women did not answer the questionnaire completely. Of the remaining 92 women, 91 women answered the dominance test correctly which indicates that the participant women understood the DCE task well and data were included in the analyses.

The demographic characteristics of the population are shown in Table II. The mean age of the women was 30.24 years. Eighty-nine percent of the women were intermediate or highly educated.

**Table II:** Patient characteristics of responders at inclusion^a^.

**Characteristics**	
Mean age in years Median duration of subfertility in months Native language, *n* (%) Dutch Other Highest level of education^b^, *n* (%) Low Moderate High Household income per year^c^, *n* (%) Low Moderate High Not reported Current fertility treatment, *n* (%) OI with or without IUI IVF or ICSI Normogonadotrophic ovulation disorder (PCOS) *n* (%) Ovulatory women with an increased OHSS risk *n* (%) Side effects previous fertility treatment, *n* (%) Yes^d^ No (Imminent) OHSS in previous cycle *n* (%) Previous cancellation treatment cycle *n* (%) Cancellation following OI cycle Cancellation following ovarian stimulation as part of IVF Parity, *n* (%) 0 1 2 or more Current status, *n* (%) Pregnant Not pregnant	30.24 (range 21–39) 8 (range 0–55) 88 (96.7%) 3 (3.3%) 10 (11.0%) 40 (44.0%) 41 (45.1%) 3 (3.3%) 35 (38.5%) 43 (47.3%) 10 (11.0%) 50 (55.0%) 41 (45.1%) 54 (59.3%) 37 (40.7%) 55 (60.4%) 36 (39.6%) 19 (20.9%) 34 (37.4%) 19 (20.9%) 15 (16.5) 64 (70.3%) 20 (22.0%) 7 (7.7%) 11 (12.1%) 80 (87.9%)

OI: ovulation induction, PCOS: polycystic ovary syndrome.

^a^Responders, *n* = 91.

^b^Low = primary school/intermediate vocational education. Moderate = Higher general secondary education/pre-university secondary education. High = Higher vocational education/university.

^c^Monthly family income of the couples was categorized according to the level of the Dutch modal income in Euros (33 000 euro): below modal, modal, above modal

^d^Most reported side effects were headache and stomach ache

### Attribute defining the choice of treatment

The results of the multinominal regression model are shown in Table III. All attributes were found to be important to the respondents and contributed to the stated choice. OHSS risk, costs, chance of pregnancy and cycle cancellation risk showed a linear effect with women’s preference. Number of injections had a non-linear relation with women’s preference and was therefore presented as ordered categories. The stated choices of the women all pointed to a preference for lower levels of the five attributes studied, as all betas had a negative sign. Concerning risk of OHSS and having cancelled cycles, this negative value means that women want to prevent both OHSS and cancellations; for chance of pregnancy, this means that women prefer a treatment that fulfils their child-wish faster, and for number of injections and costs this means that women prefer to be treated with the least number of injections at the lowest price possible.

**Table III:** Multinominal regression analysis and two latent class analyses.

**Attributes**	**Multinominal regression**		**Latent class 1** **46%**		**Latent class 2** **54%**	
	**Coeff.**	**95% CI**	**Coeff.**	**95% CI**	**Coeff**	**95% CI**
Intercept	−5.51		−3.67		−4.64	
OHSS per 1% (0–10%)	−0.22	−0.26 to −0.17	−0.075	−1.11 to −0.039	−0.36	−0.41 to −0.21
Cancellations per 1% (0–15%)	−0.086	−0.103 to −0.069	−0.19	−0.13 to −0.25	−0.051	−0.005 to −0.097
30% chance of pregnancy (1–4 treatments)	−0.84	−0.94 to −0.75	−1.41	−1.88 to −0.94	−0.55	−0.02 to −1.08
Number of injections						
0	Ref.	−	Ref.	−	Ref.	−
4	−0.52	−0.75 to −0.30	−0.31	−0.61 to 0.01	−0.87	−1.05 to −0.59
20	−0.93	−1.15 to −0.71	−0.55	−0.96 to −0.14	−1.50	−2.23 to −0.87
Costs (per 1000 euro)	−1.16	−1.21 to −1.06	−0.67	−1.09 to −0.26	−1.63	−1.98 to −1.28
2 log likelihood	−645		−601			
Pseudo*R*^2^	0.403		0.418			
cAIC^*^	1549		1397			

^*^cAIC, consistent Akaike info criterion, Coeff: coefficient,

The two most important attributes appeared to be the risk of OHSS (per 1%, mean coefficient −0.22 [95% CI −0.26–0.17]) and costs of the treatment (per 1000 euro, mean coefficient −1.16 [95% CI −1.21–1.06]), while the least important attributes were cancellation and number of injections.

We performed an unplanned sensitivity analysis excluding 11 women that conceived following treatment with gonadotrophins and were pregnant while completing the questionnaire. The resulting multinominal logit model resulted in comparable betas with a similar direction of effect.

### Preference heterogeneity

With the help of LCA, we were able to identify two subgroups of women who were dissimilar in their stated preferences. The women in latent class I (46% of all women) considered chance of pregnancy to be more important than women in latent class II (54% of all women) (−0.19 (95% CI −0.13 to −0.25) versus −0.051 (95% CI −0.005 to −0.097)). Women in latent class II considered prevention of OHSS and lower costs more important than women in latent class I (−0.075 (95% CI −1.11 to −0.039) versus −0.36 (95% CI −0.41 to −0.21). Coefficients per attribute for both subgroups are shown in Table III. We were not able to distinguish these two classes on the basis of female age and having had previous cancelled cycles (*P* = 0.63 and 0.79, respectively).

### Trade-off between OHSS and other attributes

The decrease in OHSS risk required for women to accept an increased level of another undesirable attribute was calculated. We found that women were willing to accept 5% higher risk on cycle cancellation if the OHSS rate drops 2%. Women were willing to accept one extra treatment for a reduction of 3.9% in OHSS risk. Concerning costs, women were willing to pay €1000 instead of no costs for a decrease in OHSS rate of 5.4% (Table IV).

**Table IV:** Marginal rate of substitution—the trade-off between OHSS and other attributes.

	**% decrease in chance of OHSS to accept the undesirable attribute**	
**Attribute**	**Level**	**Overall (95% CI** ^*^ **)**
Cancellation	5% more	2.1 (0.1–4.1)
Chance of pregnancy	One treatment extra	3.9 (0.6–7.2)
Number of injections	4 versus 0	2.6 (0.1–5.1)
	20 versus 0	4.2 (0.2–8.2)
Costs	€1000 versus no costs	5.4 (0.1–10.7)

^*^CI interval was based on the Krinsky–Robb method adjusted for class probabilities.

## Discussion

In the present DCE, we examined preferences of patients with an increased risk of OHSS on characteristics of fertility treatment and their evaluation of risks, burden, benefits and costs. We found that all five selected attributes played a significant role in women’s preferences for treatment. About half of the women considered chance of pregnancy to be more important, while the other half considered prevention of OHSS and lower costs to be more important. Women were willing to trade off burden, costs and pregnancy chance for lower risks on OHSS.

A strength of our study is that the design was based on the checklist of the report of the ISPOR Conjoint Analysis Experimental Design Good Research Practices Task Force (Reed Johnson *et al*. 2013). Another strength is that we focussed on women with an increased risk of OHSS and solely included this specific patient population. Furthermore, 98.9% of the women answered the dominance test correctly, which suggests that women had no difficulties understanding the questionnaire.

The main limitation of the present study concerns the sample size. The relatively small sample size made it impossible to perform several subgroup analyses. A valuable addition would be to investigate if baseline variables affect women’s choice. As pre-determined, we only investigated the effect of age and previous cycle cancellation and found no association of these variables on treatment preferences. Having had a previous cancelled cycle was in this population the result of overstimulation and/or risk for OHSS. Most of our respondents were highly educated, earning an average or high income which may have influenced their choice. In future studies it would be of value to study the effect of having experienced OHSS of different severities. Clearly, this would require a very large sample size. Furthermore, a study with in-depth interviews of women who experienced (serious) OHSS with admission to the hospital could give more insight into their considerations and the trade-offs they make.

Another limitation concerns the estimate for the number of treatment cycles required to conceive that was based on pregnancy chance following IVF and IVM. Success rates of IVM still vary a lot, but did increase over the past years (Junk and Yeap, 2012; Walls *et al*., 2015; Ho *et al*., 2018). To our knowledge, no previous research has assessed the preferences of women with an increased risk of OHSS for characteristics of IVF treatment. Two preference studies were performed among normogonadotrophic women with an ovulation disorder with regard to first- and second-line treatment. One study focused on preferences and trade-offs for laparoscopic electrocautery of the ovaries relative to OI with rFSH in clomiphene citrate-resistant women, where preferences seemed to be dominated by their effectiveness and safety (Bayram *et al*., 2005). Furthermore, Weiss *et al*. (2017) investigated the treatment preferences of women treated with OI with or without IUI. They found that women differed in their treatment preference; half of them based their preference on the lowest burden and half of them on the highest effectiveness (Weiss *et al*., 2017). Our results emphasize that effectiveness, i.e. number of treatments until conception, is not the sole important issue in fertility care. We feel that the data generated by this patient preference study provide more insight into the considerations women at risk of OHSS take into account when choosing IVF treatment. This knowledge may be used by professionals in counselling women in choosing IVF treatment and possibly in the development of decision aids.

In summary, in women with an increased risk of OHSS, the chance of OHSS is a strong attribute in determining their preference for IVF treatment and about half of the women in this study were willing to trade off burden, costs and pregnancy chance for lower risks of OHSS.
